# A Divergence-Based Approach for the Identification of Atrial Fibrillation Focal Drivers From Multipolar Mapping: A Computational Study

**DOI:** 10.3389/fphys.2021.749430

**Published:** 2021-12-24

**Authors:** Michela Masè, Alessandro Cristoforetti, Maurizio Del Greco, Flavia Ravelli

**Affiliations:** ^1^Laboratory of Biophysics and Translational Cardiology, Department of Cellular, Computational and Integrative Biology – CIBIO, University of Trento, Trento, Italy; ^2^Institute of Mountain Emergency Medicine, EURAC Research, Bolzano, Italy; ^3^Division of Cardiology, Santa Maria del Carmine Hospital, Rovereto, Italy; ^4^CISMed – Centre for Medical Sciences, University of Trento, Trento, Italy

**Keywords:** atrial fibrillation, mapping, signal processing, computational models, vector field analysis, conduction velocity, focal activity, wave propagation patterns

## Abstract

The expanding role of catheter ablation of atrial fibrillation (AF) has stimulated the development of novel mapping strategies to guide the procedure. We introduce a novel approach to characterize wave propagation and identify AF focal drivers from multipolar mapping data. The method reconstructs continuous activation patterns in the mapping area by a radial basis function (RBF) interpolation of multisite activation time series. Velocity vector fields are analytically determined, and the vector field divergence is used as a marker of focal drivers. The method was validated in a tissue patch cellular automaton model and in an anatomically realistic left atrial (LA) model with Courtemanche–Ramirez–Nattel ionic dynamics. Divergence analysis was effective in identifying focal drivers in a complex simulated AF pattern. Localization was reliable even with consistent reduction (47%) in the number of mapping points and in the presence of activation time misdetections (noise <10% of the cycle length). Proof-of-concept application of the method to human AF mapping data showed that divergence analysis consistently detected focal activation in the pulmonary veins and LA appendage area. These results suggest the potential of divergence analysis in combination with multipolar mapping to identify AF critical sites. Further studies on large clinical datasets may help to assess the clinical feasibility and benefit of divergence analysis for the optimization of ablation treatment.

## Introduction

Atrial fibrillation (AF) is the most common arrhythmia in the clinical practice, with increasing prevalence due to population aging and high morbidity associated to a fivefold increase in the risk of stroke (Fuster et al., 2006; Virani et al., 2021). The most promising approach for AF treatment is represented by catheter ablation, which performs targeted lesions on the atrial surface aiming to isolate arrhythmia sources and to interrupt critical activation pathways. Following the seminal work of Haissaguerre et al. (1998), pulmonary veins (PVs) isolation has become the cornerstone of AF ablation procedures and the common approach to treat patients with paroxysmal and persistent AF. However, given the ineffectiveness of the sole PVs ablation, especially in persistent AF, novel methodologies and approaches have been proposed to identify and ablate AF drivers located outside the PVs (Stiles et al., 2018; Buist et al., 2021; Parameswaran et al., 2021; Quintanilla et al., 2021). In parallel with the expanding role of catheter ablation, novel mapping strategies have been developed to guide the procedure and improve efficacy (Mahida et al., 2014). Multipolar mapping catheters, such as the PentaRay catheter, have been introduced to guide substrate modification and to identify extra-PV foci. These systems allow reduced mapping times and greater spatiotemporal resolution. In addition, the temporal and directional information provided by the simultaneous multisite electrograms allows, in principle, the reconstruction of activation patterns during AF.

Despite the variety of signal analysis techniques available for the point-by-point analysis of single electrograms (Nollo et al., 2008; Ravelli and Masè, 2014; Baumert et al., 2016; Almeida et al., 2018, 2021; Baher et al., 2019), fewer approaches have been proposed for the analysis of simultaneous multisite electrograms and the characterization of propagation patterns. These comprise, for instance, techniques based on computation of conduction delays and wave directions (Ganesan et al., 2015, 2018, 2019), cosine model fitting (Weber et al., 2010, 2011; Roney et al., 2019), probabilistic interpolation (Coveney et al., 2020), and physics-informed neural network (Sahli Costabal et al., 2020) applied to activation time series, as well as multivariate approaches based on causality analysis applied to atrial electrograms (Richter et al., 2011; Rodrigo et al., 2016; Alcaine et al., 2017; Luengo et al., 2019; Handa et al., 2020; Masè et al., 2020).

The present study introduces a novel framework for the reconstruction of wave activation patterns and the identification of focal drivers from clinically available multipolar mapping systems. The method is based on a radial basis function (RBF) interpolation approach (Franke, 1982; Fornefett et al., 2001), which reconstructs the activation patterns in the mapping area from scattered multisite activation time series. Propagation pattern properties are quantitatively characterized by an analytical determination of the conduction velocity (CV) vector fields, providing information on conduction heterogeneity and slow conduction areas. Finally, focal activation patterns are localized by the analysis of the vector field divergence, which marks the presence of centrifugal propagation from a localized source. After presenting the methodology, the capability of the method to accurately reconstruct activation patterns and CV fields and to identify focal drivers is tested in two different simulation models. RBF reconstruction of various propagation patterns and localization of focal activity is evaluated in a tissue patch cellular automaton (CA) model (Lammers et al., 1991; Masè et al., 2005), where the reliability of the procedure is tested against electrogram loss and activation time misdetection. The localization of focal drivers in a realistic AF context is then evaluated on synthetic electrograms from an anatomically realistic and ionically detailed left atrial (LA) model (Courtemanche et al., 1998; Cristoforetti et al., 2013). Finally, we show a proof-of-concept application of the method to clinical multipolar AF mapping data.

## Materials and Methods

### Conduction Velocity Vector Field Approach for the Analysis of Multipolar Electrograms

#### Reconstruction of Activation Maps by Radial Basis Function Interpolation

The reconstruction of the activation process in the mapping plane was addressed as a multivariate interpolation problem and solved by RBFs. Let’s consider a set of *N* mapping points, with positions ${\vec{X}}_{i}=\left[x_{i},y_{i}\right]$ in the 2D catheter mapping area, where *i* indicates the recording site, and the activation time series *t*_*i*_(*n*) extracted from the corresponding mapping electrograms, where *n* numbers subsequent atrial beats. For each beat *n*, the task of the RBF interpolation is to determine a continuous and sufficiently differentiable interpolation function $f=f\left(\vec{X}\right),$ describing the variation of the activation time as a function of a generic 2D spatial position $\vec{X}$ = *[x, y]* (Franke, 1982). The function *f(*$\vec{X}$) must fulfill the interpolation constraints at the mapping point positions $\vec{X}$*_*i*_*, given by:


(1) $$f\left({\vec{X}}_{i}\right)=t_{i}\left(n\right)\;i=1,\ldots N$$

In the RBF approach the interpolation function *f(*$\vec{X}$) takes the form:


(2) $$f\left(\vec{X}\right)=\sum_{i=1}^{N}\alpha_{i}R\left(||\vec{X}-{\vec{X}}_{i}||\right)$$

where $R\left(||\vec{X}-{\vec{X}}_{i}||\right)$ are radially symmetric functions, centered on the mapping points $\vec{X}$*_*i*_*, $||\vec{X}-X_{i}||$ is the Euclidean distance between interpolation and mapping points, and α_*i*_ are the weights of the RBF base elements.

From condition (1), it follows that:


(3) $$f\left({\vec{X}}_{i}\right)=\sum_{j=1}^{N}\alpha_{j}R\left(||{\vec{X}}_{i}-{\vec{X}}_{j}||\right)=t_{i}fori=1,\ldots ,N$$

Equation 3 can be written in matrix form as:


$$[\begin{array}{cc} \begin{array}{cc} \begin{array}{c} R\left(||{\vec{X}}_{1}-{\vec{X}}_{1}||\right)\\ R\left(||{\vec{X}}_{1}-{\vec{X}}_{2}||\right) \end{array}\begin{array}{c} R\left(||{\vec{X}}_{2}-{\vec{X}}_{1}||\right)\\ R\left(||{\vec{X}}_{2}-{\vec{X}}_{2}||\right) \end{array} & \\ \begin{array}{c} \vdots \\ R\left(||{\vec{X}}_{1}-{\vec{X}}_{N}||\right) \end{array}\begin{array}{c} \vdots \\ R\left(||{\vec{X}}_{2}-{\vec{X}}_{N}||\right) \end{array} & \end{array} & \end{array}$$

(4) $$\begin{array}{cc} \begin{array}{cc} \begin{array}{c} \ldots \\ \ldots \end{array}\begin{array}{c} R\left(||{\vec{X}}_{N}-{\vec{X}}_{1}||\right)\\ R\left(||{\vec{X}}_{N}-{\vec{X}}_{2}||\right) \end{array} & \\ \ldots \begin{array}{c} \vdots \\ R\left(||{\vec{X}}_{N}-{\vec{X}}_{N}||\right) \end{array} & \end{array} & \end{array}][\begin{array}{c} \begin{array}{c} \alpha_{1}\\ \alpha_{2} \end{array}\\ \begin{array}{c} \vdots \\ \alpha_{N} \end{array} \end{array}]=[\begin{array}{c} \begin{array}{c} t_{1}\\ t_{2} \end{array}\\ \begin{array}{c} \vdots \\ t_{N} \end{array} \end{array}]$$

Or in compact form:


(5) Rα=t,

where **R** is a real-symmetric N × N matrix and α and **t** are N × 1 vectors.

It is sometimes useful to add a low order polynomial term to the interpolant function in Eq. 2 to gain polynomial precision for some portions of *f* (e.g., to reproduce linear and constant parts of the function) and to ensure solvability of the interpolation problem.

Defining *p*_*j*_, *j* = 1, 2, …, *M* as a basis of the polynomial space and adding it to Eq. 2, we obtain the following expression for the interpolant function:


(6) $$f\left(\vec{X}\right)=\sum_{i=1}^{N}\alpha_{i}R\left(||\vec{X}-{\vec{X}}_{i}||\right)+\sum_{j=1}^{M}\beta_{j}p_{j}\left(\vec{X}\right)$$

with additional constraints for the polynomial part (Fornefett et al., 2001):


(7) $$\sum_{i=1}^{N}\alpha_{i}p_{j}\left({\vec{X}}_{i}\right)=0,j=1,\ldots ,M$$

Adding the polynomial in the interpolant function and considering these extra-constraints in Eq. 7 leads to the linear system of equations:


(8) $$\left[\begin{array}{cc} \text{R} & \text{P}\\ {\text{P}}^{T} & 0 \end{array}\right]\left[\begin{array}{c} \alpha \\ \beta \end{array}\right]=\left[\begin{array}{c} \text{t}\\ 0 \end{array}\right]$$

where **P** is a N × M matrix and **P***^T^* indicate the transposed form of **P**.

It can be demonstrated that with proper choice of the RBFs and of the polynomial term, the left-hand side matrix in Eq. 8 is non-singular and thus the system of equations is solvable and unique values for α and β can be determined (Fornefett et al., 2001; Kybic et al., 2002a,b).

In the present study, the Duchon’s radial cubic function was used as basis:


(9) $$R_{i}\left(\vec{X}\right)={\left(||\vec{X}-{\vec{X}}_{i}||\right)}^{3}$$

and a first-order polynomial term was added to the interpolant function:


(10) $$P\left(\vec{X}\right)=\beta_{1}+\beta_{2}x+\beta_{3}y$$

Interpolation with Duchon’s functions has an elegant theory in a Hilbert space setting, where Eqs 6–8 were derived as the solution of a variational problem targeting minimization of Duchon’s semi-norm and the interpolant curvature (Duchon, 1977). Duchon’s functions were shown to display excellent accuracy when interpolating scattered data, visual pleasantness and smooth appearance, low complexity, and reduced computational and memory costs (Franke, 1982). In addition, in contrast to multiquadratic or Gaussian RBFs they do not require the subjective choice of additional tuning parameters (Franke, 1982).

#### Analytical Determination of Conduction Velocity Vector Fields

Conduction velocity vector fields were analytically computed from the RBF reconstructions of the activation process $f\left(\vec{X}\right)$. The interpolant function $f\left(\vec{X}\right)$ describes activation as a function of position and sections of the function at constant time describes local isochronal contours. The gradient vector ∇⁡*f*, whose components are given by the partial derivatives of $f\left(\vec{X}\right)$:


(11) $$\nabla f=\left[\frac{\partial f}{\partial x},\frac{\partial f}{\partial y}\right]$$

is, by definition, normal to isochrone contours and thus it defines the direction of wavefront propagation (i.e., it is parallel to the velocity vector).

The components of the 2D velocity vector $\vec{v}=\left[v_{x},v_{y}\right]$ are given by:


(12) $$\begin{array}{c} v_{x}=\frac{dx}{dt}=\frac{\partial x}{\partial t}+\frac{\partial x}{\partial y}\frac{\partial y}{\partial t}\\ v_{y}=\frac{dy}{dt}=\frac{\partial y}{\partial t}+\frac{\partial y}{\partial x}\frac{\partial x}{\partial t} \end{array}$$

As detailed in Bayly et al. (1998), Eq. 12 can be solved by assuming that the direction of propagation is specified by the normal to the isochronal contours (i.e., the direction of propagation is parallel to the gradient in Eq. 11), resulting in the following relationship for the two velocity components:


(13) $$v_{y}=\frac{\frac{\partial f}{\partial y}}{\frac{\partial f}{\partial x}}v_{x}$$

Combining Eqs 11–13 an expression for velocity estimates can be obtained, which is directly linked to the partial derivatives of the interpolant function:


(14) $$\begin{array}{c} v_{x}=\frac{dx}{dt}=\frac{\frac{\partial f}{\partial x}}{{\left(\frac{\partial f}{\partial x}\right)}^{2}+{\left(\frac{\partial f}{\partial y}\right)}^{2}}\\ v_{y}=\frac{dy}{dt}=\frac{\frac{\partial f}{\partial y}}{{\left(\frac{\partial f}{\partial x}\right)}^{2}+{\left(\frac{\partial f}{\partial y}\right)}^{2}} \end{array}$$

Velocity estimates can be analytically computed through Eq. 14, once $f\left(\vec{X}\right)$ has been determined (i.e., once α and β have been calculated from Eq. 8). This means that the computation of the velocity vector field requires no additional manual operations with respect to the determination of an activation map.

#### Localization of Focal Drivers by Divergence Analysis

Focal activation sites are defined as sites or regions, which centrifugally activate the surrounding atrial tissue (Haissaguerre et al., 1998). CV vector fields corresponding to centrifugal activation present well-defined angular properties, resulting in positive divergence values. To identify focal activation, the divergence operator was applied to the analytically determined CV vector field. Before divergence computation, CV vectors were normalized to unit vectors $\vec{v}=\left[v_{x},v_{y}\right]$, to consider the sole contribution of vector angular properties. The divergence (*D*) of the vector field _$\vec{v}$_ on the mapping plane in Cartesian coordinates is given by:


(15) $$D=\nabla \cdot \vec{v}=\frac{\partial v_{x}}{\partial x}+\frac{\partial v_{y}}{\partial y}$$

where _∇⋅_ represents the divergence operator. *D* yields a signed scalar with positive values in presence of field sources and negative values for field sinks. Focal activation sites are thus located in correspondence of the local maxima of D.

### Validation of Radial Basis Function Framework by Computer Simulations

#### Validation on a Simulated Tissue Patch

The capability and accuracy of the method to reconstruct activation patterns, quantify propagation properties, and detect focal activation sites were evaluated in a bidimensional tissue patch CA model (Lammers et al., 1991; Masè et al., 2005), where the method was tested against the effects of missing electrograms and activation time misdetections.

**FIGURE 1:**
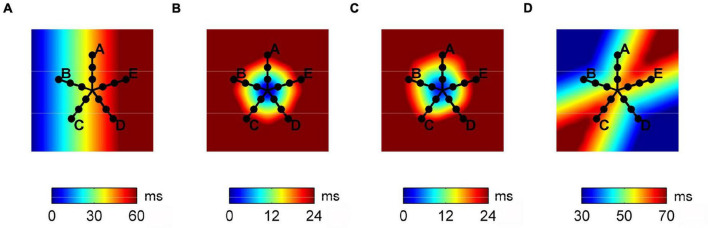
Activation maps of the propagation patterns simulated on the tissue patch cellular automaton model and position of the simulated multipolar catheter. From left to right, plane wave propagation **(A)**, focal propagation in tissues with homogeneous **(B)** and heterogeneous **(C)** conduction properties, and wave collision **(D)**.

A previously detailed bidimensional CA model of excitable tissue was used to simulate basic propagation patterns (Lammers et al., 1991; Masè et al., 2005). The model consisted of a bidimensional patch of 1000 × 1000 cell units (4 cm × 4 cm), each assigned with an evolving excitation state. As shown in Figure 1, four basic activation patterns were simulated: planar wave propagation (a), focal propagation in a tissue with homogeneous (b) and heterogeneous (c) conduction properties, and wavefront collision (d). Conduction properties were homogeneous and isotropic in patterns (a), (b), and (d) with CV values reported in Table 1. In pattern (c), eight areas with different normally distributed conduction properties were created around the focal site, resulting in a CV of 59.9 ± 12.5 cm/s. The simulation output consisted of the activation time series at each cell element, which were down-sampled on a 200 × 200 element grid to limit grid artifacts on propagation patterns. Multipolar activation time series were thus acquired from 15 mapping points, corresponding to the position of the electrode bipoles in a PentaRay catheter-like configuration, as displayed in Figure 1.

**TABLE 1:** Effects of electrogram removal on the estimation of median velocity, velocity vector magnitudes, and directions.

Simulated pattern	Removed electrograms	Exact median speed (cm/s)	Estimated median speed (cm/s)	Absolute single value speed error (%)	Absolute angle error (rad)
Plane wavefront (pattern a)	0	60.4	60.4	0.16	0.002
	2	60.4	60.4 (60.4, 60.4)	0.16 (0.14, 0.18)	0.002 (0.001, 0.002)
	4	60.4	60.4 (60.4, 60.4)	0.18 (0.15, 0.24)	0.002 (0.001, 0.002)
	6	60.4	60.4 (60.3, 60.4)	0.21 (0.16, 0.27)	0.002 (0.001, 0.002)
	8	60.4	60.4 (60.4, 60.5)	0.18 (0.14, 0.24)	0.002 (0.001, 0.003)
	10	60.4	60.4 (60.3, 60.5)	0.17 (0.12, 0.27)	0.002 (0.001, 0.003)
Focal source in homogeneous tissue (pattern b)	0	54.6	54.2	7.6	0.059
	2	54.6	53.6 (53.0, 54.1)	7.9 (7.5, 8.5)	0.065 (0.061, 0.067)
	4	54.6	53.2 (52.5, 54.2)	9.3 (8.3, 10.4)	0.074 (0.068, 0.081)
	6	54.6	54.8 (53.1, 56.6)	12.7 (10.2, 14.4)	0.098 (0.083, 0.144)
	8	54.6	59.3 (56.1, 63.1)	16.7 (14.7, 19.2)	0.167 (0.142, 0.202)
	10	54.6	66.7 (63.4, 74.6)	25.7 (20.3, 38.3)	0.265 (0.210, 0.376)
Focal source in heterogeneous tissue (pattern c)	0	59.9	59.4	7.1	0.092
	2	59.9	58.7 (57.7, 59.4)	7.8 (7.0, 8.5)	0.097 (0.093, 0.098)
	4	59.9	58.2 (56.9, 59.2)	9.2 (8.2, 10.0)	0.101 (0.096, 0.108)
	6	59.9	59.1 (56.8, 60.9)	12.0 (10.4, 13.6)	0.121 (0.106, 0.150)
	8	59.9	61.5 (57.5, 66.1)	17.0 (14.1, 19.7)	0.170 (0.140, 0.196)
	10	59.9	70.2 (63.0, 79.4)	25.2 (19.3, 36.1)	0.255 (0.201, 0.396)
Colliding wavefronts (pattern d)	0	55.8	56.6	11.4	0.088
	2	55.8	56.7 (56.4, 58.8)	12.3 (11.6, 13.1)	0.102 (0.092, 0.115)
	4	55.8	57.8 (55.9, 59.7)	13.7 (12.7, 15.4)	0.130 (0.110, 0.158)
	6	55.8	59.7 (55.1, 63.7)	18.7 (15.3, 22.5)	0.190 (0.145, 0.272)
	8	55.8	64.6 (59.4, 74.7)	23.6 (18.4, 33.1)	0.352 (0.208, 0.675)
	10	55.8	73.3 (64.3, 87.5)	31.2 (22.5, 53.2)	1.015 (0.679, 1.402)

*Data are median (IQR) over 100 stochastic repetitions.*

The accuracy of the reconstruction of CV fields was determined in the four propagation scenarios by comparing exact and estimated pointwise CV vector magnitudes and directions on the 200 × 200 grid. The localization of the focal source was evaluated in simulated scenarios (b) and (c), calculating the cell-distance between the exact position of the focal source and the maximal divergence site identified by the algorithm. The localization was considered accurate for average distances less than a distance threshold *r* = 2.7 mm (equivalent to 13.5 cells in the down-sampled 200 × 200 grid). The threshold value *r* was determined based on a statistical principle, so that the ratio between the circular area of radius *r* and the circular area swept by the simulated catheter was equal to 0.05. This corresponded to a probability <0.05 of locating the source by chance.

A stability analysis was led to test the method against factors that might corrupt clinical mapping data. The effect of electrogram loss (e.g., due to poor catheter displacement or inadequate contact) was evaluated by performing the analysis when removing a progressively larger number of randomly selected mapping points. The method stability against activation time misdetections was tested by adding random jitters to the simulated activation times series. Jitters were uniformly distributed around zero with distribution amplitude ε, varying from 0 to 20% (step 0.5%) of the activation cycle length (150 ms). The stochastic procedure was repeated 100 times for each number of sites removed and noise level. For the assessment of source localization, the catheter center was randomly moved over the patch at different repetitions.

#### Validation on an Anatomically Realistic Left Atrial Model

**FIGURE 2:**
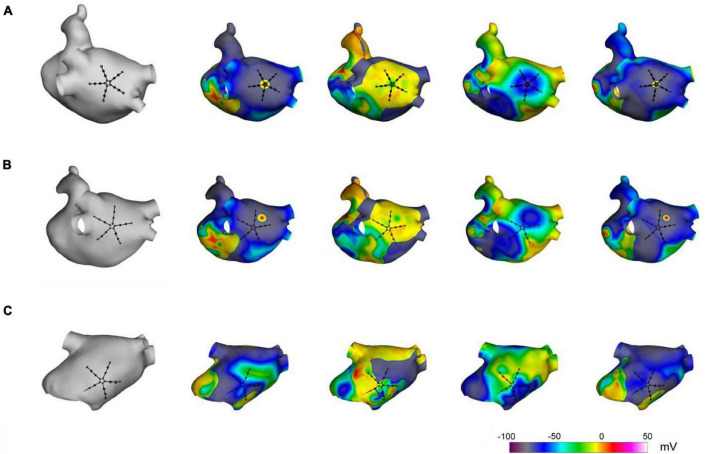
Simulation of atrial fibrillation in a realistic left atrial model. In gray, anatomical model of the left atrium and positions of the simulated multipolar catheter. In color, sequential snapshots of the membrane voltage at three mapping sites: at the focal driver **(A)**, at the boundary between the focal driver and multiple wavelets region **(B)**, and in the multiple-wavelets region **(C)**.

The capability of the method to localize AF focal drivers was tested on synthetic electrograms, obtained from an anatomically realistic LA model, based on Courtemanche–Ramirez–Nattel (CRN) cell formulation (Courtemanche et al., 1998; Cristoforetti et al., 2013). Ionic dynamics were described by the CRN human atrial cell model in monodomain formulation (Courtemanche et al., 1998). The ionic model was implemented on a realistic LA anatomy, segmented from cardiac tomography images (Cristoforetti et al., 2008). A remodeled version of the CRN model (Jacquemet et al., 2003) with an isotropic diffusion tensor of 0.2 cm^2^/s was used to obtain spiral breakups and multiple wavelet formation. After stabilization of the multiple wavelet pattern, a localized focal driver was activated in the region of the PVs. The resulting pattern comprised a centrifugal propagation in proximity of the focal driver (Figure 2, upper panels) combined with a more complex propagation with transient rotors and colliding wavefronts in the region dominated by multiple wavelets (Figure 2, lower panels).

ODE–PDE system integration was performed by a fully adaptive multi-resolution algorithm (Cristoforetti et al., 2013), which dynamically restricted computation to a set of active nodes. Reaction and diffusion were integrated with time step Δ*t* = 0.1 ms, using the Rush Larsen non-standard finite difference forward Euler method and explicit node-centered finite difference stencils (Jacquemet and Henriquez, 2005), respectively.

Synthetic electrograms were generated according to the current source approximation (Jacquemet et al., 2003) and acquired at different locations of the LA, using a PentaRay catheter configuration (see Figure 2). Specifically, 20 recording electrodes were arranged in five splines (with interelectrode distance of 4 mm), located at 0.5 mm from the atrial surface, and bipolar electrograms were computed as differences between neighboring unipolar electrograms on the same spline. Simulated signals of 5 s length, sampled at 1 kHz, were used for method evaluation.

### Proof-of-Concept Application to Clinical Mapping Data

Conduction velocity vector field reconstruction and divergence analysis were applied to clinical electrograms, retrospectively available from one patient with persistent AF, who underwent a pre-ablation electrophysiological study. The study was approved by the local Ethical Committee and performed in accordance with the principles outlined in the Declaration of Helsinki. The patient gave written informed consent. During the electrophysiological study, a 20 pole PentaRay mapping catheter (Biosense Webster, Inc., Diamond Bar, CA, United States), composed of five radiating splines, each carrying four electrodes was sequentially moved in the LA. Twenty-one atrial regions were mapped in the patient, sampling the PVs and LA body areas. Three hundred and fifteen atrial electrograms (i.e., 15 bipolar electrograms × 21 sites) of 2 s length were recorded during the study and exported for off-line analysis. Electrograms with inadequate signal-to-noise ratio were excluded from subsequent analysis. Activation time series were automatically extracted from each bipolar electrogram as previously reported (Faes et al., 2002; Masè et al., 2015). Briefly, electrograms were pre-processed to remove ventricular interference, local atrial activation waves were identified by signal filtering and adaptive threshold crossing (Faes et al., 2002; Masè et al., 2015) and atrial activation times were estimated by measuring the barycenter of local activation waves (Faes et al., 2002).

### Statistical Analysis

Data are expressed as mean ± standard deviation (SD) or median [interquartile range (IQR)], as appropriate. Divergence values are given as median, maximal, minimal, and/or range values, as appropriate.

## Results

### Conduction Velocity Vector Field Representation of Propagation Patterns

**FIGURE 3:**
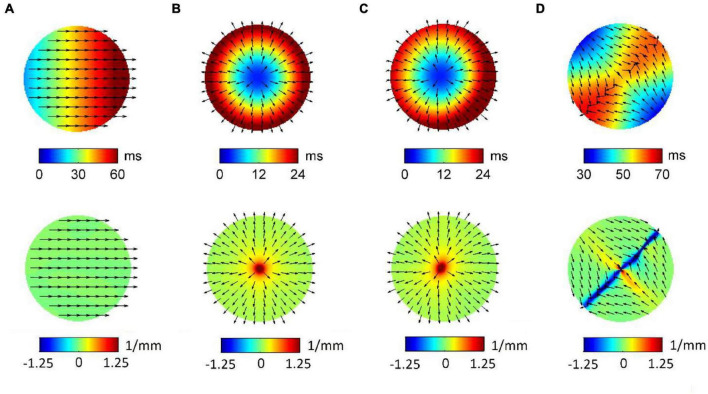
Radial basis function-reconstructed activation (top) and divergence maps (bottom) corresponding to the simulated patterns in Figure 1. Analytically determined conduction velocity vector fields are displayed on the maps as normalized arrows indicating the direction of wave propagation. Divergence maps quantify pattern properties, assigning zero divergence values (green) to planar wave propagation **(A)**, positive divergence values (red) to focal sources **(B,C)**, and negative divergence values (blue) to collision lines **(D)**.

Figure 3 displays RBF reconstructions (top panels) and superimposed normalized CV fields (arrows) corresponding to the simulated patterns in Figure 1. The angular properties of the fields are quantified by divergence maps (bottom panels). All propagation patterns were precisely reconstructed by RBF interpolation. CV vectors, which were analytically determined by RBF approach, clearly indicated the direction of wavefront propagation, being orthogonal to isochronal lines. CV values estimated from RBF reconstructions approximated well the set values, resulting of 60.4, 54.2, 59.4, and 56.6 cm/s for patterns (a) to (d). Propagation pattern properties were quantitatively distinguished in terms of divergence analysis. Indeed, planar wave propagation (a) was characterized by almost-zero values of the divergence [range (−7.5⋅10^–3^, 7.5⋅10^–3^ mm^–1^), in green]. Focal sites (b and c), acting as sources of the field, were marked by maximal positive divergence values (*D*_*max*_ = 10 mm^–1^, in red) versus the almost-zero values of the surrounding area (*D*_*median*_ = 0.125 mm^–1^). The collision line, acting as a field sink, displayed negative divergence values (*D*_*min*_ = −5.5 mm^–1^, in blue, versus *D*_*median*_ = 6.25⋅10^–3^ mm^–1^).

### Stability Analysis

The results of the stability analysis are summarized in Tables 1, 2, where reconstruction errors of CV vector magnitudes and directions are reported for the four patterns at the progressive removal of mapping sites (Table 1) and at increasing noise in activation time detection (Table 2). Reliable estimations of CV magnitudes and directions were obtained even with reduced electrogram sets (Table 1), although the number of sites necessary for the reconstructions increased with pattern complexity. The reconstruction of the plane wave pattern was not affected by the progressive removal of the electrograms. Focal patterns in homogeneous/heterogeneous tissues were reconstructed from a minimum of nine sites (six sites removed) with pointwise CV magnitude errors ∼12% and direction errors of ∼0.12 rad. Wavefront collision pattern were reconstructed from a minimum of 11 sites (four sites removed) with magnitude errors of ∼14% and direction errors of ∼0.13 rad. Median CV estimates were less affected than pointwise velocities by the reduction of sites, with percentage errors of 0.2 and −1.4% for focal and of 3.5% for collision patterns.

**TABLE 2:** Effects of temporal noise (expressed as percentage of atrial cycle length) on the estimation of median velocity, velocity vector magnitudes, and directions.

Simulated pattern	Noise level (%)	Exact median speed (cm/s)	Estimated median speed (cm/s)	Absolute speed error (%) 1 beat	Absolute angle error (rad) 1 beat	Absolute speed error (%) 10 beats	Absolute angle error (rad) 10 beats
Plane wavefront (pattern a)	0	60.4	60.4	0.16	0.002	0.158	0.002
	1	60.4	60.2 (59.8, 60.9)	3.6 (3.1, 4.3)	0.037 (0.030, 0.044)	1.4 (1.2, 1.6)	0.014 (0.012, 0.016)
	2	60.4	59.7 (58.7, 60.8)	6.9 (6.1, 8.2)	0.072 (0.060, 0.088)	2.8 (2.4, 3.2)	0.027 (0.024, 0.032)
	5	60.4	57.3 (55.4, 59.8)	16.9 (15.0, 19.0)	0.190 (0.160, 0.225)	7.0 (6.2, 8.0)	0.072 (0.065, 0.082)
	10	60.4	51.1 (47.7, 55.5)	28.3 (25.1, 33.0)	0.353 (0.292, 0.410)	16.4 (14.3, 18.0)	0.143 (0.121, 0.173)
	15	60.4	43.1 (40.0, 47.3)	37.1 (33.1, 41.2)	0.488 (0.423, 0.613)	28.1 (25.8, 30.0)	0.223 (0.188, 0.253)
	20	60.4	37.4 (34.0, 39.9)	44.2 (40.9, 48.6)	0.677 (0.527, 0.814)	38.4 (35.8, 40.5)	0.299 (0.258, 0.354)
Focal source in homogeneous tissue (pattern b)	0	54.7	54.2	7.6	0.059	7.6	0.059
	1	54.7	54.5 (53.7, 55.2)	8.7 (7.9, 9.4)	0.078 (0.073, 0.085)	7.7 (7.5, 8.0)	0.064 (0.062, 0.066)
	2	54.7	54.6 (53.3, 56.5)	11.7 (10.4, 13.1)	0.103 (0.094, 0.111)	8.4 (7.8, 8.6)	0.073 (0.070, 0.077)
	5	54.7	54.5 (51.5, 57.9)	21.5 (18.5, 24.3)	0.160 (0.142, 0.179)	10.5 (9.1, 11.3)	0.094 (0.085, 0.099)
	10	54.7	50.5 (44.8, 56.2)	32.3 (27.5, 36.0)	0.273 (0.228, 0.319)	14.0 (12.4, 15.9)	0.122 (0.106, 0.133)
	15	54.7	43.9 (39.2, 48.7)	38.6 (34.2, 43.1)	0.381 (0.311, 0.482)	22.2 (18.9, 24.0)	0.176 (0.145, 0.197)
	20	54.7	37.8 (33.2, 42.6)	44.2 (38.9, 47.5)	0.488 (0.389, 0.661)	30.4 (27.9, 32.6)	0.213 (0.182, 0.241)
Focal source in heterogeneous tissue (pattern c)	0	59.9	59.4	7.1	0.092	7.1	0.092
	1	59.9	58.7 (57.7, 59.9)	8.3 (7.8, 9.1)	0.102 (0.098, 0.104)	7.3 (7.1, 7.6)	0.095 (0.093, 0.097)
	2	59.9	58.3 (56.7, 60.7)	11.3 (10.0, 12.8)	0.115 (0.108, 0.124)	7.9 (7.4, 8.4)	0.099 (0.096, 0.103)
	5	59.9	57.1 (52.8, 59.3)	20.8 (17.9, 23.4)	0.168 (0.154, 0.193)	10.2 (8.9, 11.2)	0.112 (0.103, 0.123)
	10	59.9	52.2 (46.7, 56.5)	33.9 (29.2, 37.4)	0.276 (0.232, 0.326)	16.0 (14.1, 18.8)	0.135 (0.116, 0.156)
	15	59.9	43.5 (40.2, 48.9)	39.8 (36.9, 44.9)	0.390 (0.319, 0.519)	25.6 (21.8, 27.9)	0.166 (0.148, 0.190)
	20	59.9	38.9 (33.3, 42.6)	46.1 (41.4, 50.1)	0.521 (0.401, 0.699)	33.8 (31.4, 36.3)	0.207 (0.175, 0.243)
Colliding wavefronts (pattern d)	0	55.8	56.6	11.4	0.088	11.4	0.088
	1	55.8	56.4 (55.7, 57.0)	12.2 (11.7, 12.8)	0.096 (0.088, 0.107)	11.3 (11.1, 11.5)	0.089 (0.085, 0.093)
	2	55.8	56.0 (54.6, 57.4)	14.2 (13.1, 15.0)	0.116 (0.102, 0.130)	11.4 (11.0, 11.7)	0.093 (0.086, 0.098)
	5	55.8	55.8 (53.3, 58.8)	19.3 (17.5, 22.0)	0.230 (0.199, 0.266)	12.9 (12.0, 14.0)	0.114 (0.103, 0.125)
	10	55.8	49.6 (45.3, 54.1)	28.8 (25.7, 31.1)	0.428 (0.350, 0.518)	18.0 (16.0, 19.7)	0.172 (0.150, 0.200)
	15	55.8	42.2 (38.6, 47.4)	36.2 (31.5, 40.6)	0.590 (0.483, 0.688)	26.3 (24.0, 28.6)	0.253 (0.209, 0.289)
	20	55.8	37.1 (33.3, 42.4)	43.4 (38.1, 47.1)	0.701 (0.563, 0.835)	35.5 (32.9, 38.0)	0.337 (0.287, 0.408)

*Data are median (IQR) over 100 stochastic repetitions.*

Activation time misdetection affected the reconstruction of all the patterns (Table 2), with a progressive increase of the estimation errors at increasing levels of noise. Noise had more severe effects on the estimation of CV vector magnitudes than on median CV estimates. Absolute errors on CV vector magnitudes for focal source patterns raised from 7.6 and 7.1% at 0% noise to 32.3 and 33.9% at 10% noise, and for wavefront collision patterns errors raised from 11.4 to 28.8%. Median CV values underestimated true CV values at a progressively higher extent with increasing noise levels. At 10% noise, median CV estimates decreased to 50.5 and 52.2 cm/s (error of −7.5 and −12.8%) in focal patterns and to 49.6 cm/s (error of −11.1%) in the wavefront collision pattern. In terms of CV vector directions, angle errors for the simulated patterns increased from a range of 0.002–0.09 rad at 0% noise levels to 0.27–0.43 rad at 10% noise. Noise effects on vector magnitudes and directions were significantly reduced by averaging CV values over few beats. At 10% noise amplitude, a 10-beat average reduced CV magnitude errors to 14–16% for focal patterns, and to 18% for the collision pattern, keeping direction estimation errors <0.18 rad in all patterns.

**FIGURE 4:**
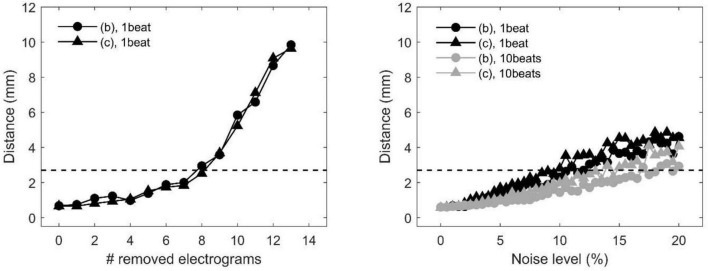
Effects of missing or corrupted data on the accuracy of localization of focal drivers by divergence analysis in a tissue patch with homogeneous (pattern b) and heterogeneous conduction properties (pattern c) in the presence of partially corrupted data. Distance between exact and estimated focal driver position as a function of the progressive removal of electrograms (left) and at increasing levels of noise in activation time series (right). The horizontal dotted line indicates the threshold for accurate localization. In the right panel, black and gray lines correspond to single-beat and 10-beat position estimation, respectively. Data are median over 100 stochastic repetitions.

The precision of divergence analysis to locate focal sources in tissues with homogeneous and heterogeneous conduction properties is reported in Figure 4 for changing number of recording sites (left) and noise levels (right). The localization strategy was stable against a reduction in the number of recording sites. Accurate identification was maintained with a minimum of nine sites available (i.e., seven sites removed). Focal drivers were precisely localized from single-beat divergence maps in presence of mild levels of noise (<11 and <10%, for homogeneous and heterogeneous conduction properties, respectively). Accurate identification at higher levels of noise (<17 and <14%) could be accomplished by averaging divergence maps over 10 beats (gray lines).

### Identification of Focal Drivers in Simulated Atrial Fibrillation

**FIGURE 5:**
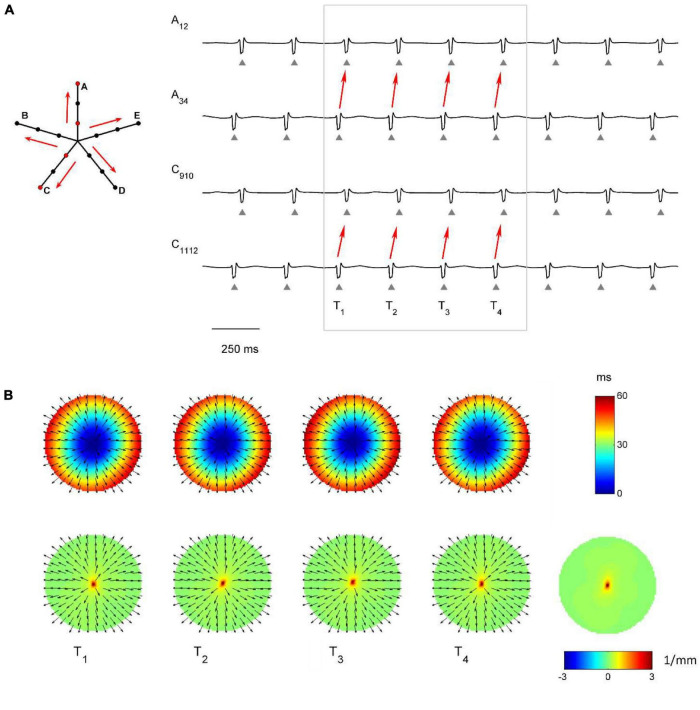
Application of divergence analysis during simulated atrial fibrillation in a region activated by a focal driver. **(A)** Schematic representation of the multipolar mapping system, indicating the central position of the bipoles, and representative synthetic electrograms corresponding to the red bipoles of the catheter. Arrows indicate the prevalent activation sequence. **(B)** Beat-to-beat reconstructed activation (top) and divergence maps (bottom) corresponding to the evidenced beats. The average divergence map over the analyzed epoch is displayed in the bottom right panel. The presence of the focal driver is evidenced by maximal positive divergence values (red).

**FIGURE 6:**
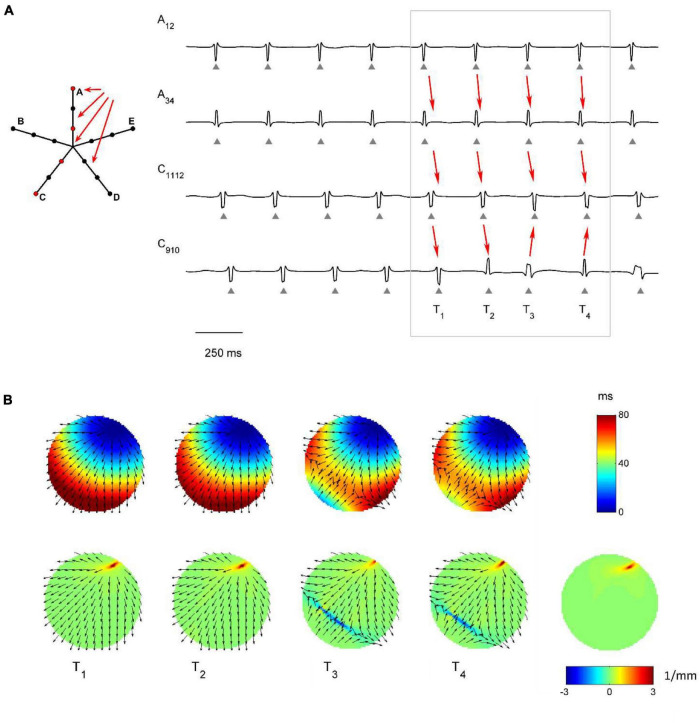
Application of divergence analysis to simulated atrial fibrillation at the boundary between the focal driver and multiple wavelets region. **(A)** Schematic representation of the multipolar mapping system, indicating the central position of the bipoles, and representative synthetic electrograms corresponding to the red bipoles of the catheter. Arrows indicate the prevalent activation sequence. **(B)** Beat-to-beat reconstructed activation (top) and divergence maps (bottom) corresponding to the evidenced beats. The average divergence map over the analyzed epoch is displayed in the bottom right panel. The presence of the focal driver is evidenced by positive divergence values (red), while collision lines are indicated by negative values (blue).

**FIGURE 7:**
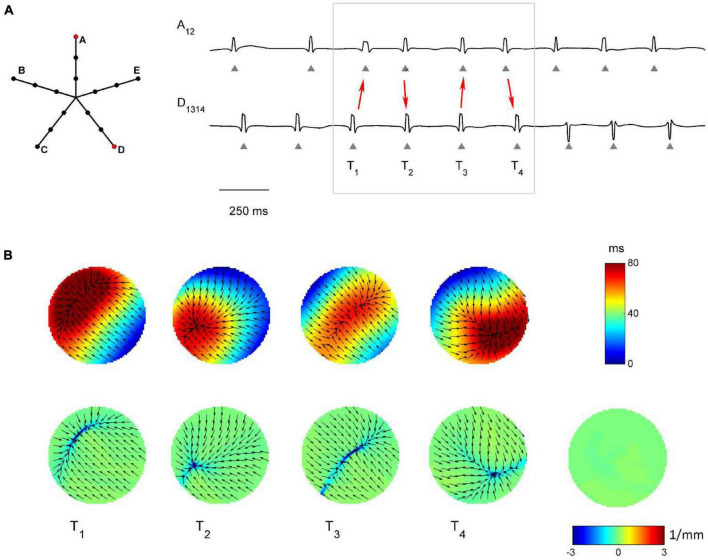
Application of divergence analysis to simulated atrial fibrillation in the region dominated by multiple wavelets propagation. **(A)** Schematic representation of the multipolar mapping system, indicating the central position of the bipoles, and representative synthetic AF electrograms corresponding to the red bipoles of the catheter. Arrows in the electrograms indicate changes in the activation sequence. **(B)** Beat-to-beat reconstructed activation (top) and divergence maps (bottom), corresponding to the evidenced beats. The average divergence map over the analyzed epoch is displayed in the bottom right panel. The absence of a stable propagation pattern is evidenced by the almost-zero values of the average divergence map (green).

The capability of the RBF framework to locate focal sources in a realistic, but controlled AF context, was evaluated by analyzing synthetic AF electrograms (Figures 5A, 6A, 7A), generated by a detailed LA model. Figures 5B, 6B, 7B show the activation and divergence maps obtained by moving the catheter from the region dominated by the focal driver (Figure 5) to the region with prevailing multiple-wavelet propagation (Figure 7).

In Figure 5 the presence of the focal driver at the center of the mapping system resulted in a centrifugal sequence of activation from internal to external recording points (i.e., from A_34_ to A_12_ and from C_1112_ to C_910_). Focal activation was apparent from the reconstructed activation maps (Figure 5B, top) and was accompanied by high positive values in the divergence maps (e.g., at time T1, *D*_*max*_ = 3.91 mm^–1^, in red, versus *D*_*median*_ = 0.10 mm^–1^). The regularity of the focal pattern could be observed comparing successive single-beat activation and divergence maps and resulted in a consistent average divergence map (*D*_*max*_ = 3.45 mm^–1^, in red, versus *D*_*median*_ = 0.10 mm^–1^).

Figure 6 displays signals and maps from an intermediate region, with the focal driver located at the top right corner of the mapping system. Here, the presence of the focal driver was less apparent from visual inspection of the recorded signals, but it was revealed by single-beat and average divergence maps, displaying maximal positive values at the focal source (e.g., in the average map, *D*_*max*_ = 3.44 mm^–1^, in red, versus *D*_*median*_ = 0.06 mm^–1^). Single-beat activation and divergence maps showed that the area was invaded by wavefronts from the multiple wavelet region, which collided with wavefronts originating from the focal driver. The presence of collision lines resulted in negative divergence values (e.g., at time T4, *D*_*min*_ = −3.02 mm^–1^, in blue, versus *D*_*median*_ = 0.07 mm^–1^).

Figure 7 shows the complex propagation patterns observed in the multiple wavelet region. The activation sequence of the simulated electrograms suggested that the area was activated by wavefronts of changing directions. This was apparent in the beat-to-beat activation and divergence maps, which showed the presence of collision lines marked by minimal negative divergence values (e.g., at time T1, *D*_*min*_ = −4.40 mm^–1^, in blue, versus *D*_*median*_ = −0.07 mm^–1^). The irregularity of the patterns and the changing position of collision lines resulted in an average divergence map with almost-zero values [range = (−0.19, 0.08) mm^–1^, in green].

### Proof-of-Concept Application to Clinical Atrial Fibrillation Data

**FIGURE 8:**
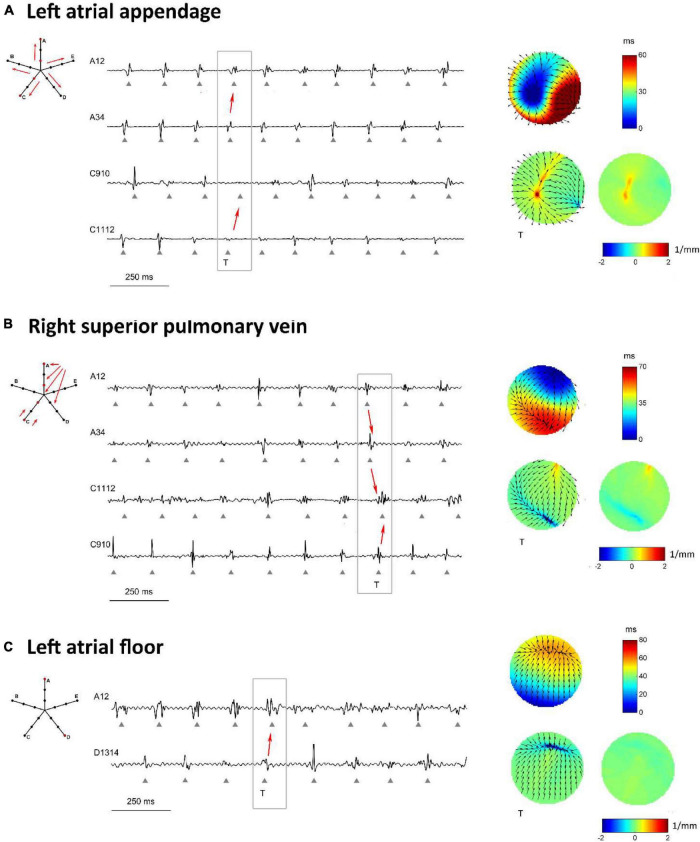
Proof-of-concept application of divergence analysis to multipolar electrograms acquired during left atrial mapping in an AF patient. The displayed multipolar mapping data were collected at the left atrial appendage **(A)**, PVs region **(B)**, and left atrial floor **(C)**. For each mapped region, the left panels show a schematic representation of the multipolar mapping system with arrows indicating the prevalent activation sequence and the representative electrograms corresponding to the red bipoles of the catheter. The right panels show single-beat reconstructed activation and divergence maps for the evidenced beat and the average divergence map calculated over the analyzed epoch.

The methodology was applied to multipolar catheter electrograms acquired in the LA of a patient with persistent AF. Three representative examples of activation and divergence maps observed in the patient in different LA regions are displayed in Figure 8. The observed patterns can be directly compared to the simulated maps of Figures 5–7.

Figure 8A displays the activation and divergence maps reconstructed from the mapping of the LA appendage region. The regular activation sequence from the internal to the external bipoles (i.e., from A_34_ to A_12_ and from C_1112_ to C_910_) and the repetitive morphology of the signals suggested the presence of a stable focal activation pattern. The reconstructed map identified the focal site at the center of the mapping area. Wavefronts propagated centrifugally from the focal site with an average CV of 44.0 ± 4.3 cm/s and activated the region at a mean cycle length of 147 ms. The stability of the focal pattern was testified by the average divergence map, which displayed a maximal positive value at the center of the mapping area (*D*_*max*_ = 1.03 mm^–1^ versus *D*_*median*_ = 0.08 mm^–1^ in the surrounding area).

The mapping of the right superior PV ostium in Figure 8B showed a regular, but more complex pattern. Activation and divergence maps suggested the presence of a focal activation site at the top corner of the mapping area (*D*_*max*_ = 0.78 mm^–1^), firing at a cycle length of 150 ± 16 ms. Wavefronts from the focal site activated the mapping area from top to bottom (i.e., from A_12_ to A_34_ to C_1112_) at an average CV of 49.8 ± 8.7 cm/s and collided with wavefronts from the bottom. Collision lines were suggested by the fragmentation of electrogram C_1112_ (Figure 8B) and were marked by negative values in the average divergence map (*D*_*min*_ = −0.82 mm^–1^).

Maps from the LA floor (Figure 8C) evidenced a complex activation process. Single-beat activation/divergence maps suggested that the region was invaded by colliding wavefronts, propagating with an average CV of 48.0 ± 4.9 cm/s. The region was characterized by an average cycle length of 160 ms and higher variability (cycle length SD of 14 ms). The average divergence map displayed almost-zero values [range = (−0.24, 0.11) mm^–1^], reflecting the instability of the activation process and the absence of a prevalent propagation pattern.

Overall, the patient’s mapping data showed an average LA cycle length of 150.1 ± 7.5 ms, with the fastest activity (136.9 ± 2.7 ms) recorded in the region of the vein of Marshall and the slowest (159.6 ± 2.9 ms) on the LA floor. Reconstructed CV vector fields showed that CV values in the LA ranged from 40.8 to 58.5 cm/s, with a mean value of 47.2 ± 4.5 cm/s. Beat-averaged divergence maps evidenced the presence of focal activation patterns in the region of the LA appendage, right superior PV and vein of Marshall, where maximal divergence values were observed (*D*_*max*_ = 1.00 ± 0.28 mm^–1^). Collision lines were observed in proximity of focal sites and in ostial regions, where average divergence maps displayed minimal negative values (*D*_*min*_ = −1.03 ± 0.64 mm^–1^). Differently, mapping sites on the LA body were prevalently characterized by complex and variable propagation patterns with more uniform divergence maps.

## Discussion

This study introduced and validated by computer simulations a novel approach for the characterization of wave propagation and the identification of focal drivers in AF, based on a RBF reconstruction of local CV vector fields from multipolar mapping electrograms. Computer simulations demonstrated the method flexibility in reconstructing continuous activation patterns and CV fields corresponding to different propagation patterns from scattered activation time series, and its accuracy in localizing focal drivers even in the presence of partially corrupted data. The proof-of-concept application to clinical multipolar mapping data detected focal activation patterns in the PVs and LA appendage region and more complex propagation patterns on the LA body, suggesting the potential of the approach for identifying critical sites in human AF.

### Radial Basis Function-Based Conduction Velocity Vector Approach for the Characterization of Propagation Patterns

Our approach was based on a RBF reconstruction of activation patterns and corresponding CV vector fields in the mapping area. The RBF approach presents several features, which makes it suitable for integration with clinically available mapping systems. RBF interpolation does not require any assumptions on the spacing and/or density of the interpolation points (Fornefett et al., 2001; Kybic et al., 2002a,b). This allows integration with different clinically available mapping systems, with respect to other approaches (Rogers et al., 1997; Bayly et al., 1998; Alcaine et al., 2014; Zeemering et al., 2020) proposed in the experimental setting, which instead require regularly spaced and/or high-density latency data. In this study we demonstrated the capability of RBFs to accurately reconstruct CV fields from the analysis of simultaneous electrograms from multipolar catheters and we showed that the application of operators, such as the divergence, to the calculated CV fields could be used to identify focal drivers in the presence of complex propagation patterns. This extends our preliminary work (Masé and Ravelli, 2010), where we suggested the possibility of using RBFs to reconstruct activation patterns and CV fields from scattered latency data, consecutively acquired by electro-anatomic mapping system, during atrial pacing. In addition, in the present work we corroborated the stability of the reconstructions at a progressive reduction of the mapping sites, suggesting that the method may be able to cope with partial information loss due to inappropriate deployment and/or poor electrode contact with the endocardial surface.

**FIGURE 9:**
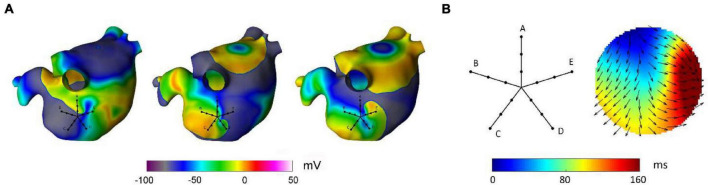
Reconstruction of a rotational pattern by radial basis functions during simulated atrial fibrillation in a realistic left atrial model. In panel **(A)**, the sequential snapshots of the membrane voltage show the presence of a transient rotor, which activates the PentaRay mapping area in a counterclockwise direction. Panel **(B)** shows the activation map reconstructed from RBF interpolation of the activation times recorded at the PentaRay bipoles, with the normalized conduction velocity vectors indicating the direction of wave propagation. The RBF activation map tracked the activation sequence, but it was not able to reproduce the head-meet-tail part of the circuit **(B)**.

A second advantage of RBF interpolation is that the methodology displays a certain degree of flexibility in reconstructing continuous activation patterns that can be present during atrial arrhythmias, such as focal activation, multiple wavelet propagation, and wave collision. This may represent an advantage with respect to previously proposed algorithms, such as cosine or ellipse model fitting (Weber et al., 2010, 2011; Roney et al., 2019) and triangulation approaches (Kojodjojo et al., 2006; Ravelli et al., 2011), which display accuracy in the estimation of wavefront speed, direction, and conduction anisotropy in the presence of a single propagating wavefront, but are not able to operate in the presence of other propagation patterns. Flexibility in reproducing different activation patterns, such as focal activation and wave collision, has been recently demonstrated by physically informed neural network, which may represent a promising approach also to quantify the epistemic uncertainty associated with these predictions (Sahli Costabal et al., 2020). Despite the flexibility of RBFs to reproduce different propagation patterns, it should be noticed that the definition of the interpolant function as a sum of continuous functions makes RBFs incapable to accurately reconstruct patterns where discontinuities and/or abrupt changes in activation time are present. Thus, although capable to reconstruct wavefronts with different curvature, RBFs may not be suitable to trace the head-meet-tail region and phase singularity of rotors, where discontinuities in phase values are present. This is exemplified in Figure 9, which displays the RBF reconstruction of a transient rotor observed in the complex activity region of the simulated AF. In the displayed time window, the electrical activity in the PentaRay mapped area was characterized by a rotational wave activating the tissue in a counterclockwise direction (Figure 9A). The RBF reconstruction (Figure 9B) was able to track the sequence of activation, showing a wave entering from the upper left region and turning to the right, but it could not reliably map the head-meet-tail part of the reentrant circuit. Given this limitation, specific techniques available in the literature, which detect rotors by examining the characteristics of the electrograms obtained from catheters (Roney et al., 2017; Ganesan et al., 2018, 2019; Orozco-Duque et al., 2019; Li et al., 2020), should be used to supplement our approach when the aim is the precise localization of rotors’ critical sites. Another pattern that may not be correctly reproduced by our method is the occurrence of conduction block. Indeed, our reconstruction algorithm assumes that activation times are correctly identified and aligned per beat at different atrial sites. In the presence of conduction block and missed activations at some space locations, direct RBFs interpolation may wrongly extrapolate a continuous electrical activation in these areas. To address this problem, in future implementations, missed activation times should be properly marked on the activation map and restrictions to interpolate activation patterns in these regions may be posed. Alternatively, imposition of late activation times at these sites may be considered to mimic conduction blocks in terms of extremely slow conduction areas.

Radial basis functions provide an analytical formulation of the activation patterns, which can be directly used for the analytical determination of the CV vector field and its properties in the mapping area. CV fields integrate and complete the information content of activation maps, providing quantitative data on local CVs and directions of propagating wavefronts. In combination with spatial and/or anatomical information, CV maps evidence spatial heterogeneities in conduction and slow conduction areas. As well, specific angular properties of CV vectors mark areas of focal activation and/or wave collision (Fitzgerald et al., 2005). Of note, in a study performing a quantitative comparison of the use of vector maps and isochrone cardiac activation maps to identify patterns and features associated with arrhythmias, the former displayed superior performance in mapping simple arrhythmias, reducing the number of measurements necessary to select the correct ablation target and presenting more rapid learning curves (Fitzgerald et al., 2004). Despite the potentiality of CV vector field representation of activation patterns (Fitzgerald et al., 2004), CV field maps have been mostly limited to experimental models (Bayly et al., 1998; Eijsbouts et al., 2003; Fitzgerald et al., 2003) and open-heart surgery settings (Hansson et al., 1998), and are only recently entering electrophysiological mapping data software (O’Shea et al., 2019; Williams et al., 2021). Our approach makes the construction of a CV vector maps equivalent to that of an activation map, in terms of both clinician skill and time expenditure, which supports clinical applications. Indeed, the analytical formulation derived from RBFs allows the automatic determination of the direction of propagation (given by the gradient of the function), solving the problem of precisely delineating the activation front. The interpolation function is globally estimated from all latency data, without need of point latency grouping as required by triangulation and bilinear fit model approaches (Fitzgerald et al., 2003; Kojodjojo et al., 2006; Ravelli et al., 2011). The potential of RBF approaches to measure CV is testified by a recent study, where RBFs were chosen to estimate CV values in an open software for mapping data analysis (Williams et al., 2021). Together with the computation of CV fields by RBFs, the present study implemented the application of operators, such as the divergence, to the CV fields. We showed the divergence able to reveal and quantify important arrhythmia features, such as the presence of focal sources or wave collision lines. These features may thus be used to inform the mapping strategy in order to accelerate the detection of these critical sites in the absence of human pattern recognition. Few previous studies have proposed algorithms to navigate the mapping catheter toward focal or early activation sites (Roney et al., 2014; Weber et al., 2017; Ganesan et al., 2019). An algorithm based on iterative regression analyses displayed high accuracy to predict the earliest activation site during focal tachycardias, requiring a significantly lower number of mapping points with respect to an operator-guided mapping (Weber et al., 2017). However, the method was developed to work in combination with a point-by-point acquisition mapping and thus with a single bipole at each catheter location, while instead our approach has the advantage of condensing in the divergence index the collective information from a multipolar catheter at each mapping location. Collective information from multipolar mapping systems was extracted in another study (Roney et al., 2014) by fitting a single planar or circular wave model to the activation times of the bipoles. The direction of the estimated wave was used to guide the catheter toward the earliest activation focal source. As our approach, this method had the advantage not to be limited to a specific catheter configuration, although better detection of focal sources was obtained in combination with PentaRay or spiral catheters than with circular catheters. While the approach in Roney et al. (2014) provided a global evaluation of the direction of propagation in the mapped area by a single-wave fit, our method allows a more local and precise description of the actual wave propagation patterns. In the absence of a source in the mapped area, local RBF-estimated CV vectors may be nonetheless easily combined in a macroscopic average vector to determine a prevalent propagation direction and thus direct the mapping process. More recently, a multiparameter algorithm was proposed to iteratively guide the incremental movement of circular catheter through the atrium until source (either focal or reentrant) detection (Ganesan et al., 2019). The detection was based on the computation of four indices describing the propagation pattern and the fulfillment of source detection criteria. In particular, focal sources were detected based on the “so-called” wave divergence index, which was approximated as the SD of the direction of the velocity vectors computed through subgrouping of the mapping points into triads (Ganesan et al., 2019). Differently from this method, our divergence approach may have the advantage of not requiring any subgroup of electrodes nor specific catheter configurations or symmetries, and of providing a divergence evaluation directly based on a physical definition. Our method may be conveniently integrated into these mapping navigation schemes, where the RBF-based detection of focal sources would complement the rules for the identification of rotors (Ganesan et al., 2019). However, the implementation would require the estimation of reliable threshold values for the divergence index, which may be determined by receiver-operating characteristic analysis or statistical approaches (Schreiber and Schmitz, 2000).

It is important to notice that our approach shares with all these methods the necessity of a correct detection of activation times. Indeed, although the use of an interpolation approach allows flexibility for pattern reconstruction, interpolation is more sensitive to activation time misdetections and noise effects, with respect to techniques based on model fitting (Bayly et al., 1998; Fitzgerald et al., 2003; Weber et al., 2010; Alcaine et al., 2014) or approaches that do not require activation time detection (Richter et al., 2011; Rodrigo et al., 2016; Alcaine et al., 2017; Luengo et al., 2019; Handa et al., 2020). In order to reduce inaccuracy in activation time estimations, activation waveforms from patient data were automatically identified by a well-established technique (Botteron and Smith, 1995; Faes et al., 2002), and activation times were set at the waveform barycenter (Faes et al., 2002; Masè et al., 2005, 2015). As suggested in several works (Holm et al., 1996; Faes et al., 2002; El Haddad et al., 2013; Ravelli and Masè, 2014), the use of a morphology-based activation detection, such as the barycenter method, improves estimation accuracy in the presence of fragmented electrograms. In addition, the barycenter identifies the central point of the activation waveform and thus can be spatially associated with the midpoint of the bipole, where bipolar electrogram coordinates were set. The importance of an accurate estimation of activation series was pointed out by our computer simulations, which showed a progressive deterioration of estimation accuracy at increasing noise levels. Noise affected at a higher extent the estimation of CV vector magnitudes, while vector directions (and thus divergence analysis) were less affected. On the other hand, median CV displayed a decreasing trend with increasing noise, which may be partially related to a minimization of the wavefront curvature performed by RBF. Since simulations showed the method to be more robust against electrogram loss than misdetection, it should be preferable to exclude activation series from noisy or excessively fragmented electrograms, although extrapolation of activation patterns to poorly mapped regions should be considered with caution. Alternatively, uncertainty in local activation times may be profitably addressed using novel approaches based on probabilistic interpolation, able to keep into account activation time errors at different sites and to pin the activation map more strongly at site with higher precision measurements (Coveney et al., 2020). Consistently with previous works (O’Shea et al., 2019), our simulations also suggested that, in the presence of stable patterns, noise effects on CV estimation and source localization could be reduced by averaging values over few beats. Thus, although the method is potentially able to work on a beat-to-beat basis, sequential divergence maps should be computed to distinguish transient instances of focal activation [e.g., due to epicardial breakthroughs (de Groot et al., 2010)] from the presence of a stable localized source, whose activity should be repetitive and persist over longer periods (Takahashi et al., 2006; de Groot et al., 2010; Ravelli et al., 2012, 2014). Assuming an atrial cycle length <200 ms during AF, averaging of 10 beats would require very short (<2 s) signal windows, which are consistent with clinical mapping times. The method may thus be used to complement other descriptors of the fibrillatory patterns, such as causality-based approaches (Richter et al., 2011; Rodrigo et al., 2016; Alcaine et al., 2017; Luengo et al., 2019; Handa et al., 2020), which may require longer signal windows for the analysis.

### Divergence-Based Identification of Focal Patterns in Human Atrial Fibrillation

The proof-of-concept application of divergence analysis to clinical multipolar electrograms revealed the presence of stable focal activation patterns at the PVs, vein of Marshall, and LA appendage during AF. The position of the detected focal sites is consistent with previous studies in AF patients (Haissaguerre et al., 1998; Hwang et al., 2000; Schmitt et al., 2002; Nitta et al., 2004; Sanders et al., 2005; Ravelli et al., 2012, 2014). Arrhythmic episodes of multifocal origin, with triggers located outside the PV region, were observed in AF patients by multisite biatrial mapping using a basket catheter or a non-contact mapping system (Schmitt et al., 2002). Rapid repetitive activity from the LA veins, including the PVs (Haissaguerre et al., 1998) and the vein of Marshall (Hwang et al., 2000), were reported to trigger paroxysmal AF. Dominant frequency analysis applied to atrial electrograms in paroxysmal AF showed that the PVs and ostial regions were most likely to harbor a high frequency source (42%), while the probability decreased in other atrial regions and in the coronary sinus (Sanders et al., 2005). In patients with permanent AF and mitral valve disease (Nitta et al., 2004), intraoperative mapping of the entire atrial epicardium showed LA focal activation from the posterior region adjacent to the PVs and the LA appendage. Consistently, electro-anatomic mapping and combined cycle length/wave similarity analysis in patients with persistent AF localized AF sources in the PV region in 47% of patients and in the LA appendage area in 12% of patients (Ravelli et al., 2012). Our analysis revealed the presence of collision areas and more complex propagation patterns in proximity of the focal sites, as observed at the right superior PV (see Figure 8B) and in other ostial regions. This is consistent with experimental (Kalifa et al., 2006) and clinical studies (Stiles et al., 2008; Ravelli et al., 2014; Kochhäuser et al., 2017), which identified zones characterized by propagation pattern variability and fractionated activity alongside areas of fast and regular atrial activation.

In the present study the analysis of clinical data was restricted to a proof-of-concept, being limited to the LA mapping of a single AF patient. Nonetheless, the consistency of our results with previous studies suggests the potential of the approach and fosters the performance of systematical studies on larger patient groups to investigate the spatial distribution of focal activation patterns in AF and their correlation with ablation outcomes. In addition, the developed techniques may be useful to map more organized forms of AF, as well as secondary atrial tachycardias at re-do procedures (Haissaguerre et al., 2006). Further validation of the method in larger clinical datasets, including different types of atrial arrhythmias, is necessary to clinically validate the method and to assess its applicability and benefit for the optimization of ablation strategies.

## Conclusion

This paper introduced a novel methodology for the characterization of wave propagation and the identification of focal drivers in AF, which is based on the reconstruction of CV vector fields and the application of divergence analysis. Tests led by computer simulations suggested that accurate reconstruction of propagation patterns and localization of focal sites was feasible with clinically available catheter configurations. The proof-of-concept application of the methodology to human AF signals consistently identified focal patterns in the PVs and LA appendage area. The combination of RBF and divergence analysis with other methods for the extraction of collective information from multipolar mapping data may allow a more robust interrogation of cardiac conduction patterns, potentially leading to the optimization of ablation treatment.

## Data Availability Statement

The datasets generated and analyzed in this study are available from the corresponding authors on reasonable request.

## Ethics Statement

The part of the study involving human participants was reviewed and approved by the Ethical Committee for Clinical Experimentation of the Provincial Agency for Health Services of the Autonomous Province of Trento. The patients/participants provided their written informed consent to participate in this study.

## Author Contributions

MM designed the study, performed the simulations and analysis, and wrote the manuscript. AC performed the simulations. MD collected the clinical data. FR designed and supervised the study, and critically revised the manuscript for important intellectual content. All authors contributed to manuscript revision, read, and approved the submitted version.

## Conflict of Interest

The authors declare that the research was conducted in the absence of any commercial or financial relationships that could be construed as a potential conflict of interest.

## Publisher’s Note

All claims expressed in this article are solely those of the authors and do not necessarily represent those of their affiliated organizations, or those of the publisher, the editors and the reviewers. Any product that may be evaluated in this article, or claim that may be made by its manufacturer, is not guaranteed or endorsed by the publisher.
